# Chronic Physical and Vicarious Psychosocial Stress Alter Fentanyl Consumption and Nucleus Accumbens Rho GTPases in Male and Female C57BL/6 Mice

**DOI:** 10.3389/fnbeh.2022.821080

**Published:** 2022-02-10

**Authors:** Daniela Franco, Andreas B. Wulff, Mary Kay Lobo, Megan E. Fox

**Affiliations:** ^1^Department of Anatomy and Neurobiology, University of Maryland School of Medicine, Baltimore, MD, United States; ^2^Department of Anesthesiology and Perioperative Medicine, Penn State College of Medicine, Hershey, PA, United States

**Keywords:** fentanyl, chronic stress, sex differences, nucleus accumbens, synthetic opioids

## Abstract

Chronic stress can increase the risk of developing a substance use disorder in vulnerable individuals. Numerous models have been developed to probe the underlying neurobiological mechanisms, however, most prior work has been restricted to male rodents, conducted only in rats, or introduces physical injury that can complicate opioid studies. Here we sought to establish how chronic psychosocial stress influences fentanyl consumption in male and female C57BL/6 mice. We used chronic social defeat stress (CSDS), or the modified vicarious chronic witness defeat stress (CWDS), and used social interaction to stratify mice as stress-susceptible or resilient. We then subjected mice to a 15 days fentanyl drinking paradigm in the home cage that consisted of alternating forced and choice periods with increasing fentanyl concentrations. Male mice susceptible to either CWDS or CSDS consumed more fentanyl relative to unstressed mice. CWDS-susceptible female mice did not differ from unstressed mice during the forced periods, but showed increased preference for fentanyl over time. We also found decreased expression of nucleus accumbens Rho GTPases in male, but not female mice following stress and fentanyl drinking. We also compare fentanyl drinking behavior in mice that had free access to plain water throughout. Our results indicate that stress-sensitized fentanyl consumption is dependent on both sex and behavioral outcomes to stress.

## Introduction

Repeated and severe stress has long been associated with the emergence of psychiatric disorders including substance use disorders (Sinha, 2008; Hollon et al., 2015; Sapolsky, 2015; Newman et al., 2018; Wemm and Sinha, 2019). Opioid use disorder (OUD) is of particular concern as rates of opioid misuse have skyrocketed, especially in North America (GBD 2016 Alcohol and Drug Use Collaborators, 2018). Accompanying the increase in OUD is an alarming number of overdose deaths and decreased average lifespan (Dowell et al., 2017). Synthetic opioids are a major contributor to increased death rates, and in 2020, caused ∼75% of drug overdose deaths in the United States (Ahmad et al., 2021; Skolnick, 2021).

A wealth of literature has sought to uncover how stress drives increased susceptibility to substance use, and the neurobiological underpinnings of comorbid stress and substance use disorders [For recent reviews see: Newman et al. (2018) and Calarco and Lobo (2020)]. Psychosocial stress is of particular interest as it is thought to more closely mimic human stressors. However, the effects of psychosocial stress on substance use are often divergent and depend on the drug, stress duration, and species [reviewed in Neisewander et al. (2012)]. For example in rats, brief social stress increases cocaine self-administration and motivation for cocaine (Haney et al., 1995; Tidey and Miczek, 1997; Covington and Miczek, 2001; Burke and Miczek, 2015), while prolonged social stress reduces cocaine self-administration (Miczek et al., 2011). In mice, prolonged social stress either promotes or suppresses cocaine self-administration (Yap and Miczek, 2007; Han et al., 2015, 2017; Arena et al., 2019) but we recently showed this depends on individual stress-response and social housing conditions (Engeln et al., 2021). There are comparatively fewer studies on psychosocial stress and opioid self-administration. In rats, social stress does not influence heroin self-administration (Cruz et al., 2011), while in mice it is associated with increased morphine preference (Cooper et al., 2017).

To investigate how stress influences vulnerability to synthetic opioid use, we adapted an oral fentanyl paradigm in male rats (Shaham et al., 1992) to male and female mice. In the Shaham model, rats were subjected to daily immobilization stress and presented with fentanyl in the homecage drinking water for 4 days (“forced consumption”). Then, rats experienced alternating periods of a choice between fentanyl and water, and additional forced consumption periods. Over time and as concentrations increased, stressed rats increased their fentanyl preference relative to unstressed rats. We sought to replicate this finding in male and female C57BL/6 mice using a similar alternating forced/choice protocol with increasing concentrations. We chose the C57BL/6 mouse since many transgenic tools are developed in this strain (Gong et al., 2007). Like Cooper et al. (2017), we used the standardized chronic social defeat stress (CSDS) procedure (Berton et al., 2006; Krishnan et al., 2007; Golden et al., 2011; Fox et al., 2020a,b). CSDS produces anhedonia in the majority of mice (∼60%, termed “stress-susceptible”), along with a decrease in motivated behaviors such as social interaction (Krishnan et al., 2007; Der-Avakian et al., 2014; Heshmati and Russo, 2015; Fox and Lobo, 2019). The other ∼40% are termed “stress-resilient” and do not display deficits in motivated behavior.

When studying stress and OUD vulnerability, it is also paramount to account for pain-related confounds (Dib and Duclaux, 1982) due the analgesic effects of opioids. Thus, to eliminate pain associated with physical injury (Cooper et al., 2017), we also used chronic witness defeat stress (CWDS), in which mice witness the social defeat of a C57BL/6 mouse and experience “vicarious” social stress (Warren et al., 2013, 2014; Iñiguez et al., 2018). Like CSDS, CWDS produces susceptible and resilient cohorts marked by decreased or unaltered social interaction and motivation, respectively. Since anhedonia and reduced motivation are associated with substance use and dependence (Hatzigiakoumis et al., 2011), we included stress-susceptibility as a factor in our analysis.

Numerous brain regions are sensitive to both drugs and stress, including the nucleus accumbens (NAc) (Hollon et al., 2015; Newman et al., 2018; Calarco and Lobo, 2020). Indeed, both chronic stress and opioid exposure cause structural changes in the NAc that are thought to drive stress-susceptibility or increased drug intake (Matsubara et al., 1999; Robinson et al., 2002; Spiga et al., 2005; Diana et al., 2006; Christoffel et al., 2011a,b; Golden et al., 2013; Pal and Das, 2013; Graziane et al., 2016; Guegan et al., 2016; Kobrin et al., 2016; Francis et al., 2017; Cahill et al., 2018; Geoffroy et al., 2019; Fox et al., 2020a,b). These structural changes are primarily driven by Rho GTPases, and we and others have shown opioid withdrawal (Cahill et al., 2018) and CSDS (Francis et al., 2017; Fox et al., 2020a) engage and alter NAc RhoA signaling. We thus examined the consequences of our combined stress and fentanyl paradigm on expression of GTPases associated with dendritic remodeling (Nakayama et al., 2000; Negishi and Katoh, 2005; Newey et al., 2005; Chen and Firestein, 2007).

Here, we test three primary hypotheses. First, that psychosocial stressors will increase fentanyl consumption in mice, Second, that stress-sensitized consumption is dependent on stress response, and Third, that fentanyl and stress exposure will alter Rho GTPase expression in the NAc.

## Materials and Methods

### Experimental Subjects

All experiments were approved by the Institutional Animal Care and Use Committee at the University of Maryland School of Medicine (UMSOM) and performed in accordance with NIH guidelines for the use of laboratory animals. Mice were given food and water *ad libitum* and housed in the UMSOM vivarium on a 12:12 h light: dark cycle. Experimental mice were 8–9 weeks old male and female C57BL/6 mice bred at UMSOM. Male CD-1 retired breeders (Charles River, >4 months) were used as the aggressors for CSDS/CWDS. Mice were randomly assigned to control or stressed groups. The forced/choice cohort (see below) included *n* = 12 unstressed males, 12 unstressed females, 23 CSDS males, 19 CWDS males, and 20 CWDS females. The choice cohort included *n* = 11 unstressed males, 13 unstressed females, 31 CSDS males, 17 CWDS males, and 15 CWDS females. We excluded 1 unstressed male mouse from this experiment due to abnormally low social interaction behavior.

### Chronic Social Stress

Social stress was performed as in our previous work (Fox et al., 2020a,b; Engeln et al., 2021; Morais-Silva et al., 2021), by using the modifications described in Warren et al. (2013) and Iñiguez et al. (2018) for vicarious resident-intruder stress. In chronic social defeat stress (CSDS), a male mouse (intruder) is physically defeated by an aggressive CD-1 (resident) for 10 min in a hamster cage containing woodchip bedding and a perforated divider. Only male mice are used in CSDS because CD-1s will not defeat female mice without modification to either the female mouse or the CD-1 (Takahashi et al., 2017; Harris et al., 2018). In chronic witness defeat stress (CWDS), a male or female mouse is housed on the opposite side of the perforated divider and allowed to witness the agonistic resident-intruder interactions for 10 min. The CSDS mouse is then housed opposite the resident, and the CWDS mouse is removed and housed opposite to a new CD-1 resident. Following 24 h of sensory interaction, the CSDS mouse is defeated by a new CD-1 resident while a different CWDS mouse witnesses the agonistic interaction. This process repeats for 10 days for 10 novel CD1-CSDS-CWDS pairings. Unstressed control mice are pair-housed across perforated dividers in cages containing woodchip bedding with a sex-matched conspecific for 10 days. Immediately following the last stressor, both stressed and unstressed mice are housed individually in woodchip bedding cages.

### Social Interaction Testing

Twenty-four hours after the last stressor, mice were tested for stress-susceptibility in a 3-chamber social preference test (Morais-Silva et al., 2021). Mice were placed in an arena (60 × 40 cm, white walls and floor) divided into three chambers (20 × 40 cm) by perforated clear acrylic dividers. The two outer chambers contain wire mesh cups, while the central chamber is empty. The experimental mouse is placed in the central chamber of the arena with two empty wire mesh cups and allowed to explore for 5 min. Then the experimental mouse is allowed to explore the arena for an additional 5 min, this time with unfamiliar sex-matched adult conspecific in one of the wire mesh cups. The amount of time spent in the chamber containing the cups (empty or novel mouse) is measured with video tracking software (TopScan Lite, CleverSys, Reston, VA, United States), and used to determine stress-phenotype. Mice spending <170 s in the mouse-paired chamber were deemed “susceptible,” and >170 s were “resilient.” This 170 s cutoff was chosen based on the average time unstressed mice spend in the mouse-paired chamber.

### Homecage Fentanyl Administration

Following social interaction testing, mice were weighed then pair-housed across a perforated divider with a sex and stress-phenotype matched conspecific in a woodchip bedding cage. Each mouse was provided two 50 mL conical tubes with rubber stoppers and ballpoint sipper tubes (Ancare, Bellmore, NY, United States). All tubes were weighed daily to determine liquid consumption, and the volume consumed was normalized to individual mouse weights.

For the first 4 days (“forced epoch 1”), both tubes contained 5 μg/mL fentanyl citrate dissolved in tap water (Cayman # 22659). On day 5, the solution in each mouse’s preferred tube was replaced with plain tap water, and the solution in the least-preferred tube was replaced with 10 μg/mL fentanyl (“choice 1”). On days 6–9 (“forced epoch 2”), both tubes contained 10 μg/mL fentanyl. On day 10, the preferred tube was replaced with plain water, and the least-preferred tube was replaced with 15 μg/mL fentanyl (“choice 2”). On days 11–14 (“forced epoch 3”), both tubes contained 15 μg/mL fentanyl. On day 15, the preferred tube was replaced with plain water (“choice 3”). On day 16, fentanyl solutions were replaced with water and mice were housed individually. Mice were then reassessed for social interaction behavior on day 18. Fentanyl preference was calculated as a percent of total liquid intake.

### RNA Isolation

Four 14-gauge NAc tissue punches per mouse were collected 24 h after the last social interaction and stored at −80°C until processing. RNA was extracted as described previously (Calarco et al., 2021) with TRIzol (Invitrogen; #15596018) and the EZNA MicroElute Total RNA kit (Omega Bio-Tek, Norcross, GA, United States; #R6831-01) with a DNase step (Qiagen, Germantown, MD, United States; #79254). RNA concentration and quality were determined with a NanoDrop 1000 spectrophotometer (Thermo Fisher Scientific). 400 ng of complementary DNA (cDNA) was synthesized using the reverse transcriptase iScript complementary DNA synthesis kit (Bio-Rad, Hercules, CA, United States; # 1708891), then diluted to a concentration of 2 ng/μL. Relative mRNA expression changes were measured by quantitative PCR using Perfecta SYBR Green FastMix (Quantabio, Beverly, MA, United States; #95072) with a Bio-Rad CFX384 qPCR system. Primer sets are available in Table 1. Fold change expression was calculated using the 2^–ddCt^ method with *Gapdh* as the reference gene. Data were normalized to the respective unstressed male or female controls.

**TABLE 1:** Primers used in qPCR.

**Limk1-F**	5′-TGG GCT AGA AGG CAG CTT TA-3′
**Limk1-R**	5′-GGG ATT CAG ATC CCT GTC AA-3′
**Rac1-F**	5′-GCC ATG TAA CGC ACC TGT AA-3′
**Rac1-R**	5′-CAA AAG CTA GTC GGC TGG TC-3′
**RhoA-F**	5′-GTG AAG CCT TGT GAA CGC A-3′
**RhoA-R**	5′-TGA AAA GGC CAG TAA TCA TAC ACT-3′
**Cdc42-F**	5′-ACC TAC CCA CAT GCA CTC AT-3′
**Cdc42-R**	5′-ACT ATT ACT GGA AGG GCA AGG A-3′
**Gapdh-F**	5′-AGG TCG GTG TGA ACG GAT TTG-3′
**Gapdh-R**	5′-TGT AGA CCA TGT AGT TGA GGT CA-3′

### Statistics

All statistical analysis was conducted in GraphPad Prism (version 9, San Diego, CA, United States) and JASP (Version 0.16)^1^. For CWDS mice, we used repeated-measures ANOVA with sex and stress-phenotype as between-subject factors, and epoch as within-subject factors. For CSDS mice, we used RMANOVA with stress-phenotype as between-subject and epoch as within-subject factors. When sphericity assumptions were violated we employed the Greenhouse–Geisser correction. *Post hoc* testing employed Holm’s correction unless noted otherwise. For gene expression analysis, we used two-way ANOVA in CWDS mice, and unpaired *t*-test for CSDS mice. To compare social interaction between the forced/choice cohort and the choice cohort, we used three-way ANOVA using sex, stress-phenotype, and future-cohort as factors. We used Pearson’s correlation to examine relationships between social interaction and fentanyl preference/consumption.

## Results

### Chronic Social Stress Increases Forced Fentanyl Consumption in Male Mice

**FIGURE 1:**
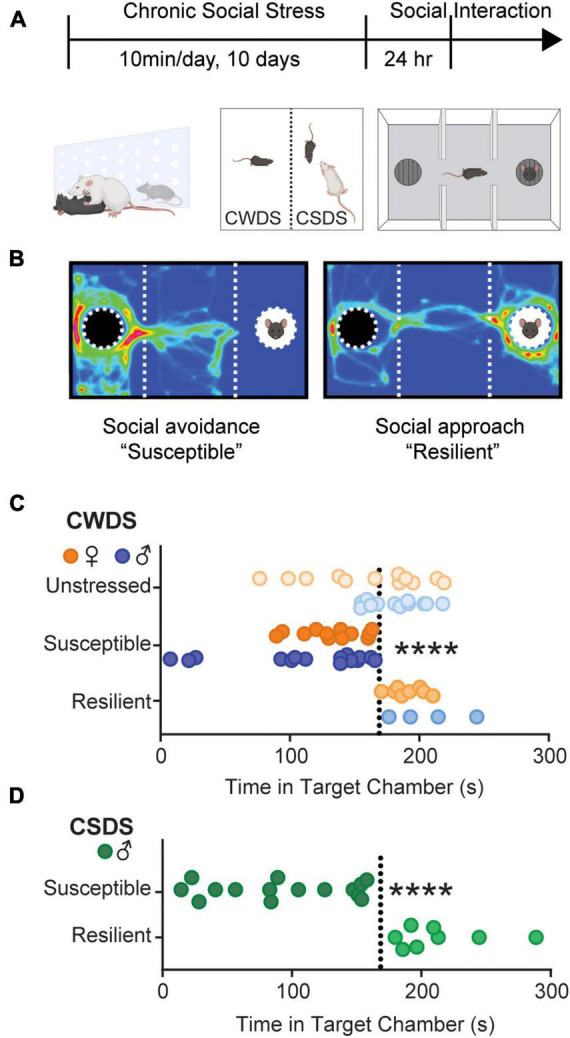
Social interaction after chronic stress. (A) Experimental timeline. Mice were physically defeated by an aggressive CD-1 resident (CSDS) or witnessed the physical defeat across a perforated divider (CWDS) for 10 days. 24 h after the last stressor, mice were assessed for stress-susceptibility in a 3-chamber social interaction apparatus. (B) Example heatmaps showing susceptible mice spend more time in the non-social compartment containing an empty cup, whereas resilient mice spend more time in the social-compartment containing a novel sex-matched conspecific. (C) Time spent in the social-compartment in unstressed, susceptible, and resilient CWDS male and female mice. Each circle represents an individual mouse. Orange circles are female, blue circles are male. (D) Time spent in the social-compartment containing a novel mouse. Each circle represents an individual mouse. CSDS mice are male only. ****p < 0.0001 compared with unstressed.

To test the hypothesis that susceptibility to psychosocial stress increases homecage opioid consumption, we subjected mice to chronic witness defeat (CWDS) or social defeat stress (CSDS) (Timeline and schematic in Figure 1A). We divided mice into subgroups based on their behavioral response to stress (Krishnan et al., 2007). Mice that displayed interaction times like unstressed mice were termed “stress-resilient” (>170 s), and those that spent less time (<170 s) were termed “stress-susceptible” (Example heat maps in Figure 1B. CWDS interaction in Figure 1C: two-way ANOVA, stress-phenotype *F*_2,57_ = 20.35, *p* < 0.0001; unstressed vs. CWDS-susceptible and CWDS-susceptible vs. CWDS-resilient, Holm-Sidak *post hoc* both *p* < 0.0001. CSDS interaction in Figure 1D: Welch’s ANOVA, *W*_2,16.51_ = 21.86, *p* < 0.0001).

**FIGURE 2:**
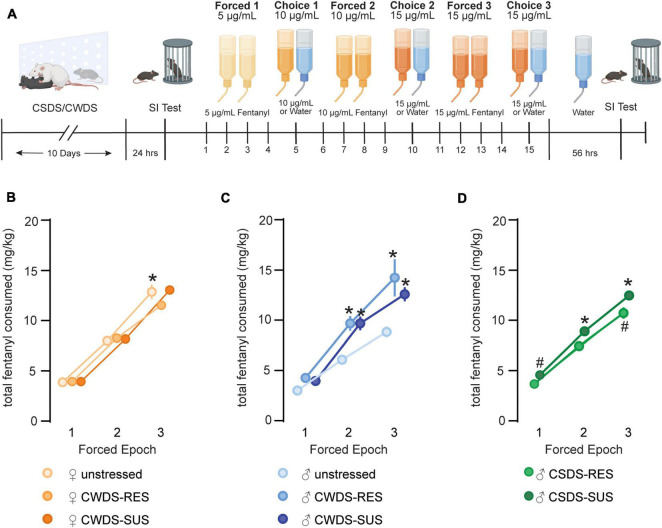
Fentanyl consumption during forced epochs. (A) Experimental timeline for forced/choice fentanyl drinking paradigm. Mice underwent 10 days of CSDS or CWDS before completing a social interaction test. Following social interaction testing, mice were organized into behavioral subgroups (susceptible vs. resilient) before starting the forced/choice fentanyl drinking paradigm. Mice experienced 3 alternating periods of forced fentanyl access and 3 periods of non-forced fentanyl access. A second social interaction test was completed ∼56 h after the last day of fentanyl access. (B) Total fentanyl consumption (mg/kg bodyweight) during forced epoch 1 (5 μg/mL), forced epoch 2 (10 μg/mL), and forced epoch 3 (15 μg/mL) in unstressed and CWDS female mice, (C) male mice, and (D) CSDS male mice. *p < 0.05, #*p* = 0.07 compared with unstressed male mice during the same epoch.

Following social interaction testing, mice were pair-housed across a perforated divider with a sex and stress-phenotype matched conspecific. Each mouse was provided two tubes containing fentanyl during the forced epochs, followed by alternating periods of fentanyl or water during the choice epochs. Over time fentanyl concentrations increased from 5 to 15 μg/mL (timeline in Figure 2A). We compared fentanyl consumption (mg/kg bodyweight) across the three forced epochs and found that as fentanyl concentrations increased, as did consumption. We found a significant effect of epoch (RMANOVA; *F*_1.7,98.7_ = 610.1, *p* < 0.001) stress-phenotype (*F*_2,57_ = 8.57, *p* < 0.001), and significant interactions (sex × stress-phenotype: *F*_2,57_ = 9.12, *p* < 0.001; epoch × sex × stress-phenotype: *F*_3.5,98.7_ = 5.94, *p* < 0.01, Figures 2B,C). After *post hoc* testing, we found no differences between subgroups in forced epoch 1. As concentration increased in forced epoch 2 and 3, CWDS males consumed more fentanyl relative to unstressed males (forced epoch 2, unstressed: 6.1 ± 1.7 vs. CWDS-susceptible: 9.7 ± 2.7 mg/kg, *p* < 0.001; vs. CWDS-resilient: 9.7 ± 1.5 mg/kg, *p* = 0.019; forced epoch 3, unstressed: 8.8 ± 1.9 vs. CWDS-susceptible: 12.6 ± 2.7 mg/kg, *p* < 0.001; CWDS-resilient: 14.2 ± 3.7 mg/kg, *p* < 0.001, Figure 2C). Stress did not influence forced consumption in female mice, as CWDS females did not consume more fentanyl relative to unstressed females in any epoch (*p* > 0.05; Figure 2B). Interestingly, we also found a sex difference that reached significance at forced epoch 3, with unstressed female mice consuming more fentanyl than unstressed males (unstressed female: 12.8 ± 2.5 vs. male: 8.8 ± 1.9 mg/kg, *p* < 0.001, Figures 2B,C).

We performed similar comparisons in CSDS mice and found a significant effect of epoch (RMANOVA, *F*_1.7,54.2_ = 494.9, *p* < 0.0001), stress-phenotype (*F*_2,32_ = 15.2 *p* < 0.001) and epoch × stress-phenotype interaction (*F*_3.4,54.2_ = 4.6, *p* = 0.005, Figure 2D). We found a trending difference between groups in forced epoch 1 with CSDS-susceptible consuming more fentanyl relative to unstressed mice (CSDS-susceptible: 4.6 ± 0.7 vs. unstressed: 3.0 ± 0.7 mg/kg, *p* = 0.07, Figures 2C,D). CSDS-susceptible mice also consumed more fentanyl during forced epoch 2 (CSDS-susceptible 8.9 ± 1.8 vs. unstressed: 6.1 ± 1.7 mg/kg, *p* < 0.001) and forced epoch 3 (CSDS-susceptible: 12.5 ± 1.9 unstressed: 8.8 ± 1.9 mg/kg, *p* < 0.001, Figures 2C,D). CSDS-resilient mice consumed more during forced epoch 3, although this failed to reach statistical significance (CSDS-resilient: 10.7 ± 1.6 mg/kg vs. CSDS-susceptible, *p* = 0.07; vs. unstressed, *p* = 0.07 Figures 2C,D). Together, this indicates that both CWDS and CSDS increased forced fentanyl consumption in stress-susceptible male mice.

### Sex and Stress-Susceptibility Influences Fentanyl Consumption During Choice Periods

**FIGURE 3:**
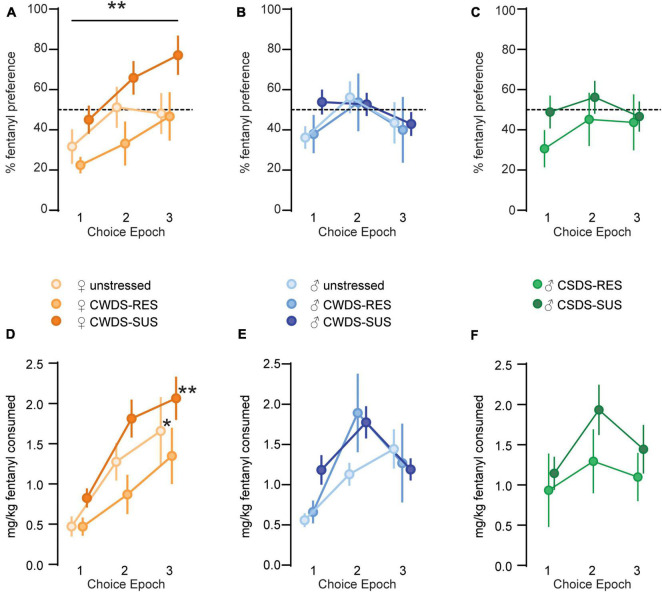
Fentanyl consumption and preference during choice epochs. (A) Fentanyl preference (%) for Choice 1 (10 μg/mL or water), 2 (15 μg/mL or water), and 3 (15 μg/mL or water) in unstressed, susceptible, and resilient female CWDS mice, (B) male mice, and (C) CSDS male mice. **p = 0.006 female choice 1 vs. female choice 3. (D) Fentanyl consumed (mg/kg bodyweight) during choice epochs in unstressed, susceptible, and resilient female CWDS mice, (E) male mice, and (F) male CSDS mice. *p = 0.01 unstressed female choice 3 vs. choice 1, **p = 0.005 CWDS-susceptible female choice 3 vs. choice 1.

On choice days, we replaced the solution in the mouse’s preferred tube with plain tap water, and their least-preferred tube with 10 or 15 μg/mL fentanyl (Timeline in Figure 2A). When we examined fentanyl preference across choice epochs, we found a main effect of epoch (*F*_1.9,111.1_ = 4.5, *p* = 0.04), stress-phenotype (*F*_2,57_ = 4.2, *p* = 0.02), and epoch × sex interaction (*F*_1.9, 111.1_ = 3.3, *p* = 0.04, Figures 3A,B). *Post hoc* analysis showed no significant differences between the subgroups during choice 1, 2, or 3, however, female mice as a whole increased preference between choice 1 and 3 (choice 1: 34.4 ± 4 vs. choice 2: 52.2 ± 6%, *p* = 0.15, vs. choice 3: 58.7 ± 6% *p* = 0.006, Figure 3A). We performed similar comparisons in physically stressed CSDS mice and found no significant effect of epoch or stress-phenotype for %fentanyl preference (Epoch: *F*_2,64_ = 2.3, *p* = 0.1; stress-phenotype: *F*_2,32_ = 0.6, *p* = 0.5, Figure 3C).

We next examined mg/kg fentanyl consumption during choice epochs and found significant effects of epoch (*F*_1.9,108.9_ = 19.3, *p* < 0.001), stress-phenotype (*F*_2,57_ = 6.1, *p* = 0.004) and an epoch × sex interaction (*F*_1.9,108.9_ = 5.7, *p* = 0.005, Figures 3D,E). Male and female mice responded differentially to the concentrations of fentanyl available during the choice periods. Similar to the % preference data, female unstressed and female CWDS-susceptible mice increased their mg/kg fentanyl consumption between choice 1 and 3 (female unstressed choice 1: 0.5 ± 0.1 vs. choice 3: 1.6 ± 0.4 mg/kg, *p* = 0.01, CWDS-susceptible choice 1: 0.8 ± 0.1 vs. choice 3: 2.1 ± 0.3 mg/kg, *p* = 0.005, Figure 3D). Neither female CWDS-resilient, nor any male-CWDS increased their consumption across choice epochs. We performed similar comparisons in physically stressed CSDS mice and found significant effects of epoch (*F*_2,64_ = 3.6, *p* = 0.03) and stress-phenotype (*F*_2,32_ = 3.5 *p* = 0.04, Figure 3F). This effect was likely driven by CSDS-susceptible mice which consumed more mg/kg fentanyl compared with unstressed mice (Overall consumption, unstressed: 2.6 ± 0.4 vs. CSDS-susceptible: 4.5 ± 0.6, *p* = 0.04, vs. CSDS-resilient: 3.3 ± 0.7, *p* = 0.4).

### Stress-Susceptibility Is Predictive of Choice Fentanyl Consumption in Chronic Witness Defeat Stress Mice

**FIGURE 4:**
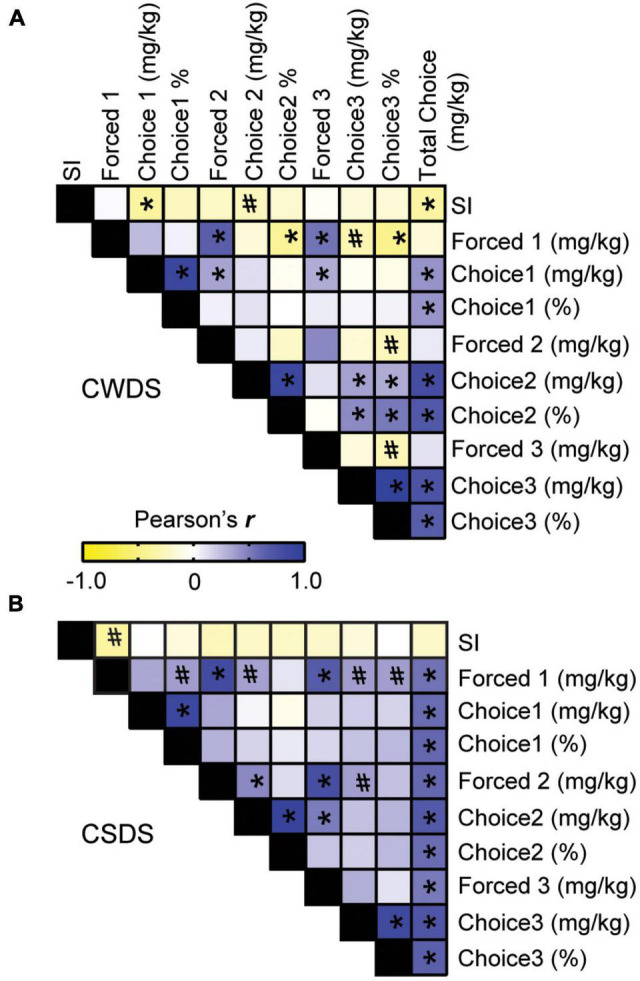
Pearson correlation matrix of stress-susceptibility and fentanyl consumption. (A) Paired correlations between stress-phenotype (measured by social interaction, SI), fentanyl consumption and fentanyl preference in CWDS mice and (B) CSDS mice. Negative correlations are in yellow; positive correlations are in blue. *p < 0.05, #*p* < 0.09.

Given that stress-susceptible mice consumed more fentanyl, we next sought to strengthen the relationship between stress-phenotype and fentanyl preference. We performed Pearson’s correlations using social interaction data from individual mice. In CWDS mice, we found a negative correlation between social interaction and mg/kg fentanyl consumed during Choice 1, indicating that the less time spent interacting with a novel conspecific (i.e., more stress-susceptible), the more fentanyl the CWDS mouse consumed during Choice 1 (Pearson’s *r* = −0.382, *p* = 0.026; Figure 4A). Similarly, we found a trending negative correlation between stress-susceptibility and mg/kg fentanyl consumed during Choice 2 (Pearson’s *r* = −0.270, *p* = 0.097; Figure 4A). By Choice 3, stress-susceptibility was no longer correlated with mg/kg fentanyl consumption in CWDS mice (*p* = 0.372, Figure 4A), however, it remained a predictor of overall choice intake (Pearson’s *r* = −0.371, *p* = 0.019). Of note, there was no significant relationship between social interaction and mg/kg fentanyl consumption or % preference in unstressed mice (not shown, *p* = 0.18). By contrast, stress-susceptibility was not significantly correlated with mg/kg fentanyl consumed during any choice period in CSDS mice (Choice 1 *p* = 0.94, Choice 2, *p* = 0.287, Choice 3, *p* = 0.484 Figure 4B), however, there was a trend toward stress-susceptibility increasing fentanyl consumption during forced epoch 1 (*r* = −0.383, *p* = 0.07, Figure 4B). The strongest relationship between choice consumption in CSDS mice was forced consumption, indicating physical dependence may play more of a role in CSDS mice (e.g., total choice and forced epoch 2, *r* = 0.60, *p* ≤ 0.001, Figure 4B).

### Abstinence From Homecage Fentanyl Increases Stress-Susceptibility Dependent on Stress-Phenotype

**FIGURE 5:**
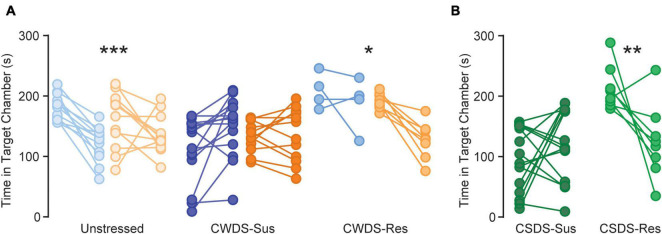
Changes in social interaction after fentanyl exposure. (A) Time in social target chamber (s) during the first social interaction (before fentanyl drinking) and second social interaction (56 h after fentanyl drinking) for unstressed, susceptible, and resilient CWDS and (B) CSDS mice ***p < 0.001, unstressed before vs. after fentanyl, *p = 0.036, CWDS-resilient before vs. after fentanyl, **p = 0.002, CSDS-resilient before vs. after fentanyl.

Opioid abstinence and withdrawal can promote anhedonia and social interaction deficits (Bai et al., 2014; Becker et al., 2017; Bravo et al., 2020). To test how our stress and fentanyl paradigm influenced social interaction, we next replaced the fentanyl solutions with plain water for 2 days and single housed mice prior to reassessing social interaction behavior. We chose this ∼56 hr time point to avoid acute withdrawal symptoms during testing. We found significant effects of stress-phenotype (RMANOVA, *F*_2,57_ = 8.4, *p* < 0.001), time (*F*_1,57_ = 11.4, *p* = 0.001), and a time × stress-phenotype interaction (*F*_2,57_ = 14.7, *p* < 0.0001; Figure 5A), but not sex (*p* = 0.1). All unstressed and CWDS-resilient groups showed reduced time interacting with a novel sex-matched conspecific relative to the first social-interaction (before vs. after fentanyl: unstressed *p* ≤ 0.001, CWDS-resilient *p* = 0.036, Figure 5A). CWDS-susceptible mice showed a nominal increase in social interaction but remained susceptible (*p* = 0.14). CSDS mice also reduced time interacting with a novel conspecific relative to the first social interaction, but this was only statistically significant in CSDS-resilient mice, possibly indicating a “floor effect” in CSDS-susceptible mice (RMANOVA, time: *F*_1,32_ = 10.4, *p* = 0.003; stress-phenotype: *F*_2,32_ = 12.90, *p* < 0.001; time × stress-phenotype: *F*_2,32_ = 8.9, *p* ≤ 0.001; before vs. after fentanyl: CSDS-susceptible *p* = 0.87; CSDS-resilient *p* = 0.002, Figure 5B). Together, this indicates fentanyl exposure and abstinence alone is sufficient to generate a stress-like phenotype.

### Chronic Stress and Fentanyl Exposure Downregulate Dendritic Complexity Molecules in the Nucleus Accumbens

**FIGURE 6:**
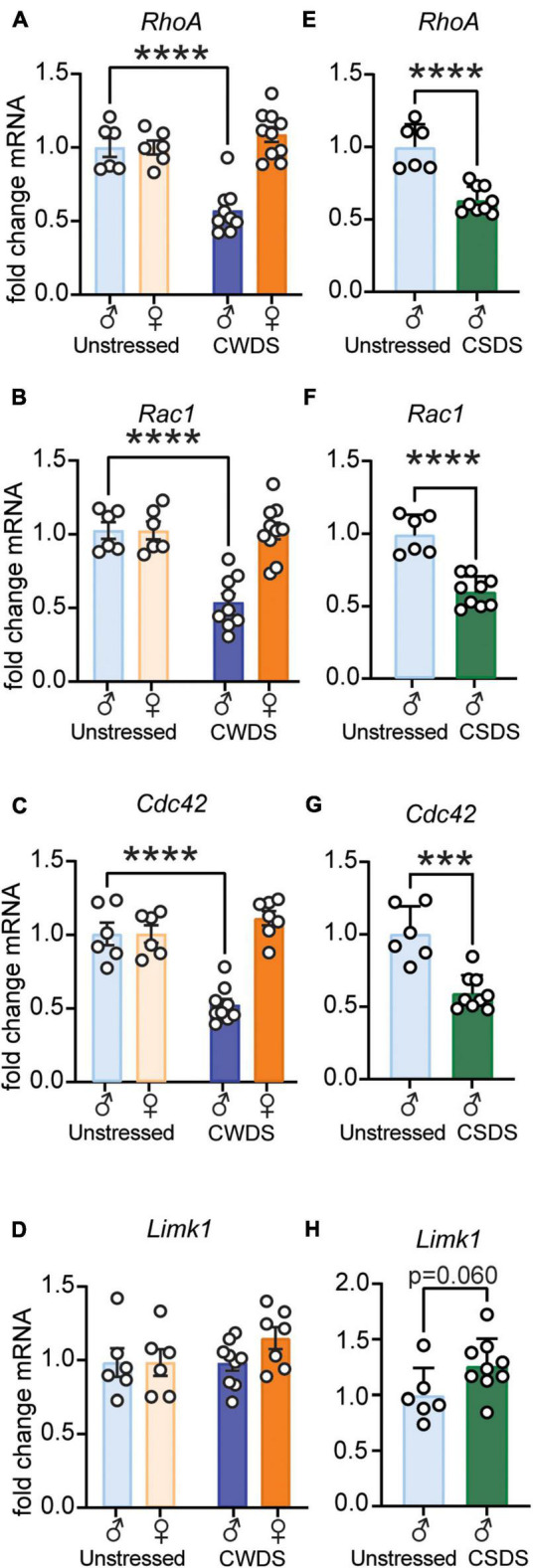
Rho GTPases RhoA, Rac1, and Cdc42 are downregulated in the Nucleus Accumbens after chronic stress and fentanyl exposure in male mice. (A–D) RhoA, Rac1, Cdc42, and Limk1 gene expression in unstressed and CWDS mice, respectively (unstressed male vs. CWDS male: *RhoA*, *Rac1, Cdc42, Limk1*, ****p < 0.0001). (E–H) RhoA, Rac1, Cdc42, and Limk1 gene expression in unstressed and CSDS mice, respectively (unstressed vs. CSDS: *RhoA, Rac1*, ****p < 0.0001; unstressed vs. CSDS: *Cdc42*, ***p = 0.0002; unstressed vs. CSDS: *Limk1*, *p* = 0.060).

To determine how stress and fentanyl altered expression of dendritic complexity molecules, we extracted NAc RNA from a subset of mice and examined expression of Rho GTPases. We found decreased expression of RhoA, Rac1, and Cdc42 in CWDS-male, but not CWDS-female mice (2-way ANOVA. *RhoA*: Stress, *F*_1,27_ = 9.36, Sex × Stress *F*_1,27_ = 21.65, Figure 6A; *Rac1*: Stress *F*_1,27_ = 16.17, Sex × Stress *F*_1,27_ = 15.70, Figure 6B; *Cdc42*: Stress *F*_1,27_ = 11.68, Sex × Stress *F*_1,27_ = 27.79, Figure 6C; all *p* ≤ 0.005. Holm-Sidak *post hoc* unstressed-male vs. CWDS-male, all *p* < 0.0001). We found similar decreased expression of Rho GTPases in CSDS mice (unpaired *t*-test. *RhoA*: t_13_ = 5.75, Figure 6E; *Rac1*: t_13_ = 6.34, Figure 6F; *Cdc42*: t_13_ = 5.14, Figure 6G; all *p* ≤ 0.0002). In contrast, we found no changes in the downstream effector Limk1 (Meng et al., 2003) in CWDS mice of either sex (2-way ANOVA, *p* > 0.05, Figure 6D). In CSDS mice we found a trend toward increased Limk1 expression (t_13_ = 2.06, *p* = 0.060, Figure 6H).

### Female Mice Exhibit Greater Fentanyl Preference in an All-Choice Paradigm

An important caveat in this work is that by forcing mice to consume fentanyl, some mice may have increased fentanyl preference due to tolerance or may consume fentanyl to mitigate withdrawal symptoms. We thus repeated the experiment in a separate cohort of mice that were given a choice between fentanyl and water for the entire experiment (“choice cohort”) As with the mice that received forced exposure (“forced/choice cohort”), we divided choice cohort mice into susceptible and resilient as described above (For CWDS, two-way ANOVA, stress-phenotype, *F*_2,50_ = 9.36, *p* = 0.0004; For CSDS, Welch’s ANOVA, *W*_2,17.43_ = 30.67, *p* < 0.0001; Table 2). Prior to the fentanyl exposure, we had a different number of mice classified as susceptible in the choice cohort as compared with the forced/choice cohort, however, there was no effect of future-cohort (three-way ANOVA, *F*_1,107_ = 0.88 *p* = 0.3516) or future-cohort × stress-phenotype (*F*_2,107_ = 2.10, *p* = 0.13) in CWDS mice, indicating the cohorts were equivalent. However, for CSDS mice, there was a future-cohort × stress-phenotype interaction (two-way ANOVA, *F*_2,71_ = 6.02, *p* = 0.0038) such that CSDS-susceptible mice in the choice cohort had greater mean social interaction time compared with mice in the forced/choice cohort (Holm-Sidak *post hoc*, *p* = 0.004). We include the data from this group in the present manuscript, but it is important to note this difference when comparing the two cohorts.

**TABLE 2:** Time spent in social target chamber before and after the fentanyl drinking paradigm in choice cohort mice.

Sex	Stress-phenotype	Time in social chamber before fentanyl	Time in social chamber after fentanyl	*N*
Male	Unstressed	166.7 ± 127.0	150.3 ± 9.5	11
	CWDS-susceptible	157.4 ± 8.5	168.9 ± 18.4	3
	CWDS-resilient	198.8 ± 5.0	176.3 ± 9.3	14
	CSDS-susceptible	137.6 ± 6.3	135.6 ± 10.2	13
	CSDS-resilient	200.0 ± 4.9	151.4 ± 7.8	18
Female	unstressed	165.6 ± 9.2	145.6 ± 12.4	13
	CWDS-susceptible	139.9 ± 7.8	131.5 ± 8.7	9
	CWDS-resilient	193.4 ± 5.0	120.4 ± 17.1	6

*CWDS-resilient mice significantly decrease social interaction time (p = 0.002).*

*CSDS-resilient mice decreased social interaction time (p < 0.001).*

Following social interaction, we pair-housed mice with a sex and stress-phenotype matched mouse. Each mouse was provided with one tube containing 5 μg/mL fentanyl and a second tube containing plain tap water for days 1–4 (“non-forced epoch 1”), 10 μg/mL fentanyl and water during days 6–9 (“non-forced epoch 2”), and 15 μg/mL fentanyl and water for days 11–14 (“non-forced epoch 3”). Tubes were rotated daily to account for side preference. We compared fentanyl consumption (mg/kg bodyweight) across the three non-forced epochs and found as fentanyl concentrations increased, as did consumption. As with the forced/choice cohort, there were sex differences in consumption: (epoch, *F*_1.8,89_ = 197.4, *p* < 0.001; sex, *F*_1,50_ = 13.5, *p* < 0.001; epoch × sex interaction (*F*_1.8,89_ = 7.7, *p* = 0.001, Table 3). We found no differences between sexes in non-forced epoch 1, however, females consume more relative to males in non-forced epoch 2 (females: 3.8 ± 0.3 vs. males: 2.7 ± 0.2, *p* = 0.038) and non-forced epoch 3 (females: 6.2 ± 0.4 vs. males: 4.0 ± 0.2, *p* < 0.001). In CSDS mice, we found only a main effect of epoch (*F*_1.7,66.9_ = 149.8 *p* < 0.001) but no differences between the subgroups, indicating that concentration, not stress, influences fentanyl consumption in these mice.

**TABLE 3:** Fentanyl consumption in choice cohort mice during non-forced epochs.

Epoch	Sex	Stress-phenotype	mg/kg fentanyl consumption	*N*
Non-forced Epoch 1	Male	Unstressed	1.2 ± 0.1	11
		CWDS-susceptible	1.3 ± 0.1	3
		CWDS-resilient	1.4 ± 0.1	14
		CSDS-susceptible	1.7 ± 0.2	13
		CSDS-resilient	1.6 ± 0.1	18
	Female	Unstressed	2.0 ± 0.3	13
		CWDS-susceptible	2.1 ± 0.2	9
		CWDS-resilient	1.9 ± 0.4	6
Non-forced Epoch 2	Male	Unstressed	2.7 ± 0.2	11
		CWDS-susceptible	2.9 ± 0.6	3
		CWDS-resilient	2.8 ± 0.3	14
		CSDS-susceptible	3.2 ± 0.4	13
		CSDS-resilient	3.2 ± 0.3	18
	Female	Unstressed	3.4 ± 0.4	13
		CWDS-susceptible	4.6 ± 0.6	9
		CWDS-resilient	3.5 ± 0.6	6
Non-forced Epoch 3	Male	CWDS-susceptible	4.1 ± 0.3	11
		CWDS-susceptible	4.5 ± 0.2	3
		CWDS-resilient	3.8 ± 0.3	14
		CSDS-susceptible	4.9 ± 0.5	13
		CSDS-resilient	4.3 ± 0.3	18
	Female	Unstressed	6.1 ± 0.6	13
		CWDS-susceptible	6.3 ± 0.7	9
		CWDS-resilient	6.1 ± 0.8	6

*Females consume more relative to males in non-forced epoch 2 (p = 0.038) and non-forced epoch 3 (p < 0.001).*

On choice days, we replaced the solution with either 10 or 15 μg/mL fentanyl as in the forced/choice cohort. When we examined fentanyl preference across choice epochs, we found only a trending epoch × sex × stress interaction (*F*_3.9,97.6_ = 2.2, *p* = 0.07) with female mice exhibiting greater % preference compared with male mice (Table 4). In CSDS mice we found only a main effect of epoch (*F*_1.9,75_ = 3.6, *p* = 0.03) such that as fentanyl concentration increased, % fentanyl preference decreased. When we examined mg/kg fentanyl consumption during choice epochs, we found a main effect of epoch (*F*_1.7,87.9_ = 34.8, *p* < 001), sex (*F*_1,50_ = 19.6, *p* < 001) and epoch × sex interaction (*F*_1.7,87.9_ = 3.4, *p* = 0.04) in CWDS mice with female mice consuming more during choice 2 (*p* < 0.001) and choice 3 (*p* = 0.005, Table 4). Like % fentanyl preference, CSDS mice showed a main effect of epoch for mg/kg consumption (*F*_1.8,70.8_ = 15.1, *p* < 0.001) with the highest consumption occurring during choice 2, regardless of stress-susceptibility (Table 4).

**TABLE 4:** Fentanyl preference and consumption in choice cohort mice.

Epoch	Sex	Stress-phenotype	% fentanyl preference	mg/kg fentanyl consumption	*N*
Choice 1	Male	Unstressed	53 ± 5%	0.7 ± 0.1	11
		CWDS-susceptible	24 ± 8%	0.4 ± 0.1	3
		CWDS-resilient	55 ± 6%	0.8 ± 0.1	14
		CSDS-susceptible	57 ± 6%	0.9 ± 0.1	13
		CSDS-resilient	58 ± 5%	0.9 ± 0.1	18
	Female	Unstressed	45 ± 6%	0.8 ± 0.1	13
		CWDS-susceptible	51 ± 8%	1.1 ± 0.2	9
		CWDS-resilient	38 ± 7%	0.8 ± 0.2	6
Choice 2	Male	Unstressed	48 ± 6%	1.3 ± 0.1	11
		CWDS-susceptible	41 ± 4%	1.0 ± 0.1	3
		CWDS-resilient	46 ± 4%	1.1 ± 0.1	14
		CSDS-susceptible	51 ± 5%	1.4 ± 0.2	13
		CSDS-resilient	44 ± 5%	1.4 ± 0.1	18
	Female	Unstressed	52 ± 5%	1.8 ± 0.2	13
		CWDS-susceptible	52 ± 7%	2.1 ± 0.2	9
		CWDS-resilient	39 ± 7%	1.7 ± 0.3	6
Choice 3	Male	Unstressed	53 ± 7%	1.1 ± 0.2	11
		CWDS-susceptible	35 ± 6%	0.7 ± 0.1	3
		CWDS-resilient	32 ± 5%	0.7 ± 0.1	14
		CSDS-susceptible	47 ± 5%	1.1 ± 0.1	13
		CSDS-resilient	39 ± 5%	1.0 ± 0.2	18
	Female	Unstressed	43 ± 4%	1.3 ± 0.2	13
		CWDS-susceptible	52 ± 5%	1.6 ± 0.2	9
		CWDS-resilient	44 ± 7%	1.3 ± 0.2	6

*Female mice consume more than male mice during choice 2 (p < 0.001) and choice 3 (p = 0.005).*

### Social Withdrawal After Fentanyl Is Maintained in Resilient Mice Despite Decreased Fentanyl Consumption

Similar to the forced/choice cohort, we retested mice for social interaction after 2 days of plain water. We found a main effect of time (RM-ANOVA, *F*_1,50_ = 8.7, *p* = 0.005), stress-phenotype (*F*_2,50_ = 3.6, *p* = 0.035), sex (*F*_1,50_ = 8.5, *p* = 0.005) and a time × stress-phenotype interaction (*F*_2,50_ = 3.6, *p* = 0.03). *Post hoc* testing showed that only CWDS-resilient mice significantly decreased time spent interacting with a novel conspecific (*p* = 0.002). In the CSDS mice, only CSDS-resilient mice decreased social interaction time (RM-ANOVA, time *F*_1,39_ = 11.4, *p* = 0.002; stress-phenotype *F*_2,39_ = 10.8, *p* < 0.001, time × stress-phenotype *F*_2,39_ = 5.0, *p* = 0.01, *post hoc*, *p* < 0.001; Table 2).

## Discussion

Here we show both physical (CSDS) and vicarious (CWDS) psychosocial stressors impact homecage opioid consumption and preference in a stress and sex-dependent manner. First, susceptibility to either CWDS or CSDS increases forced fentanyl consumption in male mice regardless of fentanyl concentration. Second, female CWDS-susceptible mice show increased fentanyl consumption during the choice periods, and greater stress-susceptibility is associated with greater fentanyl preference. However, only female CWDS-susceptible mice show increased fentanyl preference during the final choice period. Physical CSDS-susceptibility is associated with increased choice fentanyl consumption, but this effect is decoupled from social interaction behavior and more closely associated with prior forced consumption. Finally, we show CWDS and CSDS-susceptible males have downregulated Rho GTPases in the NAc.

The main goal of this study was to determine if psychosocial stress in mice produced similar effects to restraint stress in rats on fentanyl preference using the alternating periods of forced and choice consumption described by Shaham et al. (1992). Consistent with this prior work, stressed mice consumed more fentanyl than unstressed mice. In addition to physical stress, vicarious stress was sufficient to increase drug-taking, similar to Ramsey and Van Ree (1993) and Cooper et al. (2017). We also found a negative correlation between social interaction and fentanyl preference, similar to the morphine preference findings of Cooper et al. (2017). Importantly, our mice were pair-housed throughout the fentanyl procedure. Previously, we showed that when mice are single-housed, CSDS-susceptibility is associated with increased early cocaine self-administration (Engeln et al., 2021). When pair-housed, CSDS instead decreases cocaine self-administration, analogous to increased anhedonia. Consistent with our cocaine work, Alexander et al. (1978) showed socially isolated rats consume more morphine relative to socially housed rats. Thus, the small effects we report here may reflect a kind of “social buffering” that decreases opioid consumption, similar to the protective effects of social interaction on heroin and methamphetamine craving (Venniro et al., 2018, 2019). Future work should investigate how stress interacts with social housing conditions to sensitize or blunt opioid consumption.

Here we found only stress-susceptible female mice increase fentanyl preference over time. In humans, sex-differences in OUD are largely dependent on the opioid (Back et al., 2010; Lee and Ho, 2013). Recent evidence suggests that women have higher rates of prescription opioid use (Serdarevic et al., 2017) and are more likely to report using opioids to cope with negative affect relative to men (McHugh et al., 2013). Women also exhibit increased susceptibility to stress-related disorders (e.g., PTSD, depression) (Bangasser et al., 2019). While we do not see striking sex-differences in our behavioral readout of susceptibility in mice, the increased opioid consumption in our female mice mirrors that of humans and other rodents models (Alexander et al., 1978; Lynch and Carroll, 1999; Kokane and Perrotti, 2020; Hammerslag et al., 2021). In our study, female mice reliably consume more fentanyl than male mice, even when provided free choice throughout. This finding aligns well with recent work showing female mice are resistant to devaluation of oral fentanyl (Monroe and Radke, 2021), self-administer more oral oxycodone (Phillips et al., 2020), intravenous heroin (Towers et al., 2019), and remifentanil (Anderson et al., 2021). Further work is needed to establish if stress exacerbates the sex-differences in operant opioid self-administration.

When we compared male mice from our modified forced/choice model (Shaham et al., 1992) to male mice that had free-choice throughout the entire procedure, stressed males do not appear different from unstressed males. It is tempting to speculate that in the forced/choice cohort, stressed male mice exhibit increased consumption due to either tolerance that develops from the forced epoch, and/or a desire to relieve the negative affect caused by abstinence/withdrawal (Koob, 2020). Our correlation data support this interpretation, as the strongest predictor of choice intake was forced intake in CSDS mice. It is impossible to rule out that the physically stressed CSDS mice consume fentanyl to relieve pain. However, we do not believe this to be the case given the modest differences between intake in choice cohort unstressed and CSDS mice.

Social withdrawal and other anhedonia-like behaviors are commonly exhibited following opioid abstinence and withdrawal (Bai et al., 2014; Becker et al., 2017; Bravo et al., 2020). In the forced/choice cohort, all unstressed and previously resilient mice exhibited decreased social interaction; in the choice cohort, only previously resilient mice decreased social interaction. Given that choice cohort unstressed mice consumed less fentanyl as compared with forced/choice unstressed mice, this suggests the social-withdrawal is dose-dependent. Opioid abstinence in previously resilient choice cohort mice—despite the overall dose being smaller– may have been sufficient to tip them toward susceptibility. Regardless of forced/choice or choice, the CSDS-susceptible mice maintain their previous social interaction behavior, which we believe may reflect a “floor effect.” Future work examining severity of withdrawal symptoms or other stress-like behaviors could address if susceptible mice exposed to fentanyl are more or less stressed than their resilient drug-exposed counterparts.

Targeting dendritic remodeling may prove useful for blunting stress-sensitized acquisition of drug taking (Rigoni et al., 2021). Given that NAc structural plasticity is an important determinant of synaptic strength, and heavily involved in both stress and drug-related behaviors, we examined Rho GTPase expression in the NAc. Interestingly, while female mice displayed the more persistent elevation in fentanyl preference after stress, we only found differential expression in stressed male mice. Furthermore, the changes to gene expression were consistent in susceptible mice regardless of physical or vicarious stress. We found decreased expression of Rac1, RhoA, and Cdc42 in NAc of stress and force/choice male mice. This is largely consistent with decreased Rac1 expression in NAc after CSDS (Golden et al., 2013), and decreased active Cdc42 in mPFC after chronic unpredictable stress (Luo et al., 2020). Both acute morphine withdrawal (Cahill et al., 2018) and CSDS (Francis et al., 2017; Fox et al., 2020a) engage NAc RhoA signaling. Thus, the downregulated RhoA expression seen here may reflect a compensatory mechanism in males that help protects against elevated late fentanyl preference. In agreement with compensatory downregulation, Rock1, a downstream effector of RhoA, is decreased in striatum after protracted morphine withdrawal (Drastichova et al., 2021). However, our data are difficult to interpret given the competing effects of fentanyl and stress. It is also possible that the sex and stressor-dependent changes in gene expression are related to sex differences in fentanyl consumption and stress responsivity. It is also worth noting that RhoA expression and dendritic remodeling after CSDS are cell subtype specific (Fox et al., 2020a,b), and there is no work on NAc GTPases after CWDS. Future work can dissect precise sex and cell-type specific adaptations that may explain the effects of CSDS/CWDS on subsequent fentanyl consumption.

In summary, we show stress-susceptibility is associated with increased fentanyl consumption that presents differently in male and female mice. We also found sex differences in the expression of dendritic-complexity molecules in the nucleus accumbens—a region important for both stress and drug-related behaviors. Our findings here, together with our updated mouse model, will provide the basis for more precise investigations on mechanisms of stress-sensitized opioid use in both sexes of transgenic mice.

## Data Availability Statement

The original contributions presented in the study are included in the article/supplementary material, further inquiries can be directed to the corresponding author/s.

## Ethics Statement

The animal study was reviewed and approved by the University of Maryland School of Medicine IACUC.

## Author Contributions

MF designed the study. DF, AW, and MF conducted the experiments and analyzed the data. ML provided resources for conducting the experiments. MF and DF wrote the manuscript with contributions from AW and ML. All authors contributed to the article and approved the submitted version.

## Conflict of Interest

The authors declare that the research was conducted in the absence of any commercial or financial relationships that could be construed as a potential conflict of interest.

## Publisher’s Note

All claims expressed in this article are solely those of the authors and do not necessarily represent those of their affiliated organizations, or those of the publisher, the editors and the reviewers. Any product that may be evaluated in this article, or claim that may be made by its manufacturer, is not guaranteed or endorsed by the publisher.

## Footnotes

1
https://jasp-stats.org/

## References

[B1] Ahmad F. B. Rossen L. M. Sutton P. (2021). *Provisional Drug Overdose Death Counts.* Hyattsville, MD: National Center for Health Statistics.

[B2] Alexander B. K. Coambs R. B. Hadaway P. F. (1978). The effect of housing and gender on morphine self-administration in rats. *Psychopharmacology (Berl.)* 58 175–179. 10.1007/BF00426903 98787

[B3] Anderson E. M. Engelhardt A. Demis S. Porath E. Hearing M. C. (2021). Remifentanil self-administration in mice promotes sex-specific prefrontal cortex dysfunction underlying deficits in cognitive flexibility. *Neuropsychopharmacology* 46 1734–1745. 10.1038/s41386-021-01028-z 34012018 PMC8358018

[B4] Arena D. T. Covington H. E. DeBold J. F. Miczek K. A. (2019). Persistent increase of I.V. cocaine self-administration in a subgroup of C57BL/6J male mice after social defeat stress. *Psychopharmacology (Berl.)* 236 2027–2037. 10.1007/s00213-019-05191-6 30798402 PMC6626693

[B5] Back S. E. Payne R. L. Simpson A. N. Brady K. T. (2010). Gender and prescription opioids: findings from the national survey on drug use and health. *Addict. Behav.* 35:1001. 10.1016/J.ADDBEH.2010.06.018 20598809 PMC2919630

[B6] Bai Y. Li Y. Lv Y. Liu Z. Zheng X. (2014). Complex motivated behaviors for natural rewards following a binge-like regimen of morphine administration: mixed phenotypes of anhedonia and craving after short-term withdrawal. *Front. Behav. Neurosci.* 8:23. 10.3389/fnbeh.2014.00023 24550799 PMC3909833

[B7] Bangasser D. A. Eck S. R. Ordoñes Sanchez E. (2019). Sex differences in stress reactivity in arousal and attention systems. *Neuropsychopharmacology* 44:129. 10.1038/S41386-018-0137-2 30022063 PMC6235989

[B8] Becker J. A. J. Kieffer B. L. Le Merrer J. (2017). Differential behavioral and molecular alterations upon protracted abstinence from cocaine versus morphine, nicotine, THC and alcohol. *Addict. Biol.* 22 1205–1217. 10.1111/ADB.12405 27126842 PMC5085894

[B9] Berton O. McClung C. A. DiLeone R. J. Krishnan V. Renthal W. Russo S. J. (2006). Essential role of BDNF in the mesolimbic dopamine pathway in social defeat stress. *Science* 311 864–868. 10.1126/science.1120972 16469931

[B10] Bravo I. M. Luster B. R. Flanigan M. E. Perez P. J. Cogan E. S. Schmidt K. T. (2020). Divergent behavioral responses in protracted opioid withdrawal in male and female C57BL/6J mice. *Eur. J. Neurosci.* 51 742–754. 10.1111/EJN.14580 31544297 PMC7069788

[B11] Burke A. R. Miczek K. A. (2015). Escalation of cocaine self-administration in adulthood after social defeat of adolescent rats: role of social experience and adaptive coping behavior. *Psychopharmacology (Berl.)* 232 3067–3079. 10.1007/s00213-015-3947-5 25943168 PMC4515153

[B12] Cahill M. E. Browne C. J. Wang J. Hamilton P. J. Dong Y. Nestler E. J. (2018). Withdrawal from repeated morphine administration augments expression of the RhoA network in the nucleus accumbens to control synaptic structure. *J. Neurochem.* 147 84–98. 10.1111/jnc.14563 30071134 PMC6181756

[B13] Calarco C. A. Lobo M. K. (2020). Depression and substance use disorders: clinical comorbidity and shared neurobiology. *Int. Rev. Neurobiol.* 157 245–309. 10.1016/BS.IRN.2020.09.004 33648671

[B14] Calarco C. A. Fox M. E. Van Terheyden S. Turner M. D. Alipio J. B. Chandra R. (2021). Mitochondria-related nuclear gene expression in the nucleus accumbens and blood mitochondrial copy number after developmental fentanyl exposure in adolescent male and female C57BL/6 mice. *Front. Psychiatry* 12:737389. 10.3389/fpsyt.2021.737389 34867530 PMC8637046

[B15] Chen H. Firestein B. L. (2007). RhoA regulates dendrite branching in hippocampal neurons by decreasing cypin protein levels. *J. Neurosci.* 27 8378–8386. 10.1523/JNEUROSCI.0872-07.2007 17670984 PMC6673065

[B16] Christoffel D. J. Golden S. A. Dumitriu D. Robison A. J. Janssen W. G. Ahn H. F. (2011a). IκB kinase regulates social defeat stress-induced synaptic and behavioral plasticity. *J. Neurosci.* 31 314–321. 10.1523/JNEUROSCI.4763-10.2011 21209217 PMC3219041

[B17] Christoffel D. J. Golden S. A. Russo S. J. (2011b). Structural and synaptic plasticity in stress-related disorders. *Rev. Neurosci.* 22 535–549. 10.1515/RNS.2011.044 21967517 PMC3212803

[B18] Cooper S. E. Kechner M. Caraballo-Pérez D. Kaska S. Robison A. J. Mazei-Robison M. S. (2017). Comparison of chronic physical and emotional social defeat stress effects on mesocorticolimbic circuit activation and voluntary consumption of morphine. *Sci. Rep.* 7:8445. 10.1038/s41598-017-09106-3 28814751 PMC5559445

[B19] Covington H. E. Miczek K. A. (2001). Repeated social-defeat stress, cocaine or morphine: effects on behavioral sensitization and intravenous cocaine self-administration “binges.”. *Psychopharmacology (Berl.)* 158 388–398. 10.1007/s002130100858 11797060

[B20] Cruz F. C. Quadros I. M. Hogenelst K. Planeta C. S. Miczek K. A. (2011). Social defeat stress in rats: escalation of cocaine and “speedball” binge self-administration, but not heroin. *Psychopharmacology (Berl.)* 215 165–175. 10.1007/s00213-010-2139-6 21197616 PMC3707112

[B21] Der-Avakian A. Mazei-Robison M. S. Kesby J. P. Nestler E. J. Markou A. (2014). Enduring deficits in brain reward function after chronic social defeat in rats: susceptibility, resilience, and antidepressant response. *Biol. Psychiatry* 76 542–549. 10.1016/j.biopsych.2014.01.013 24576687 PMC4117827

[B22] Diana M. Spiga S. Acquas E. (2006). Persistent and reversible morphine withdrawal-induced morphological changes in the nucleus accumbens. *Ann. N. Y. Acad. Sci.* 1074 446–457. 10.1196/annals.1369.045 17105943

[B23] Dib B. Duclaux R. (1982). Intracerebroventricular self-injection of morphine in response to pain in the rat. *Pain* 13 395–406. 10.1016/0304-3959(82)90008-2 7133734

[B24] Dowell D. Arias E. Kochanek K. Anderson R. Guy G. P. Losby J. L. (2017). Contribution of opioid-involved poisoning to the change in life expectancy in the United States, 2000-2015. *JAMA* 318 1065–1067. 10.1001/JAMA.2017.9308 28975295 PMC5818798

[B25] Drastichova Z. Hejnova L. Moravcova R. Novotny J. (2021). Proteomic analysis unveils expressional changes in cytoskeleton- and synaptic plasticity-associated proteins in rat brain six months after withdrawal from morphine. *Life (Basel, Switzerland)* 11:683. 10.3390/life11070683 34357055 PMC8304287

[B26] Engeln M. Fox M. E. Lobo M. K. (2021). Housing conditions during self-administration determine motivation for cocaine in mice following chronic social defeat stress. *Psychopharmacology (Berl.)* 238 41–54. 10.1007/s00213-020-05657-y 32914243 PMC8162736

[B27] Fox M. E. Lobo M. K. (2019). The molecular and cellular mechanisms of depression: a focus on reward circuitry. *Mol. Psychiatry* 24 1798–1815. 10.1038/s41380-019-0415-3 30967681 PMC6785351

[B28] Fox M. E. Chandra R. Menken M. S. Larkin E. J. Nam H. Engeln M. (2020a). Dendritic remodeling of D1 neurons by RhoA/Rho-kinase mediates depression-like behavior. *Mol. Psychiatry* 25 1022–1034. 10.1038/s41380-018-0211-5 30120419 PMC6378138

[B29] Fox M. E. Figueiredo A. Menken M. S. Lobo M. K. (2020b). Dendritic spine density is increased on nucleus accumbens D2 neurons after chronic social defeat. *Sci. Rep.* 10:12393. 10.1038/s41598-020-69339-7 PMC7381630 32709968

[B30] Francis T. C. Chandra R. Gaynor A. Konkalmatt P. Metzbower S. R. Evans B. (2017). Molecular basis of dendritic atrophy and activity in stress susceptibility. *Mol. Psychiatry* 22 1512–1519. 10.1038/mp.2017.178 28894298 PMC5747312

[B31] GBD 2016 Alcohol and Drug Use Collaborators (2018). The global burden of disease attributable to alcohol and drug use in 195 countries and territories, 1990-2016: a systematic analysis for the Global Burden of Disease Study 2016. *Lancet Psychiatry* 5 987–1012. 10.1016/S2215-0366(18)30337-7 30392731 PMC6251968

[B32] Geoffroy H. Canestrelli C. Marie N. Noble F. (2019). Morphine-induced dendritic spine remodeling in rat nucleus accumbens is corticosterone dependent. *Int. J. Neuropsychopharmacol.* 22 394–401. 10.1093/ijnp/pyz014 30915438 PMC6545536

[B33] Golden S. A. Christoffel D. J. Heshmati M. Hodes G. E. Magida J. Davis K. (2013). Epigenetic regulation of RAC1 induces synaptic remodeling in stress disorders and depression. *Nat. Med.* 19 337–344. 10.1038/nm.3090 23416703 PMC3594624

[B34] Golden S. A. Covington H. E. Berton O. Russo S. J. (2011). A standardized protocol for repeated social defeat stress in mice. *Nat. Protoc.* 6 1183–1191. 10.1038/nprot.2011.361 21799487 PMC3220278

[B35] Gong S. Doughty M. Harbaugh C. R. Cummins A. Hatten M. E. Heintz N. (2007). Targeting Cre recombinase to specific neuron populations with bacterial artificial chromosome constructs. *J. Neurosci.* 27 9817–9823. 10.1523/JNEUROSCI.2707-07.2007 17855595 PMC6672645

[B36] Graziane N. M. Sun S. Wright W. J. Jang D. Liu Z. Huang Y. H. (2016). Opposing mechanisms mediate morphine- and cocaine-induced generation of silent synapses. *Nat. Neurosci.* 19 915–925. 10.1038/nn.4313 27239940 PMC4925174

[B37] Guegan T. Cebrià J. P. Maldonado R. Martin M. (2016). Morphine-induced locomotor sensitization produces structural plasticity in the mesocorticolimbic system dependent on CB1-R activity. *Addict. Biol.* 21 1113–1126. 10.1111/adb.12281 26179931

[B38] Hammerslag L. R. Denehy E. D. Carper B. Nolen T. L. Prendergast M. A. Bardo M. T. (2021). Effects of the glucocorticoid receptor antagonist PT150 on stress-induced fentanyl seeking in male and female rats. *Psychopharmacology (Berl.)* 238 2439–2447. 10.1007/S00213-021-05865-0 34008048 PMC10323366

[B39] Han X. Albrechet-Souza L. Doyle M. R. Shimamoto A. Debold J. F. Miczek K. A. (2015). Social stress and escalated drug self-administration in mice II. Cocaine and dopamine in the nucleus accumbens. *Psychopharmacology (Berl.)* 232 1003–1010. 10.1007/s00213-014-3734-8 25216798 PMC4339460

[B40] Han X. DeBold J. F. Miczek K. A. (2017). Prevention and reversal of social stress-escalated cocaine self-administration in mice by intra-VTA CRFR1 antagonism. *Psychopharmacology (Berl.)* 234 2813–2821. 10.1007/s00213-017-4676-8 28698920 PMC5709170

[B41] Haney M. Maccari S. Le Moal M. Simon H. Vincenzo Piazza P. (1995). Social stress increases the acquisition of cocaine self-administration in male and female rats. *Brain Res.* 698 46–52. 10.1016/0006-8993(95)00788-R 8581502

[B42] Harris A. Z. Atsak P. Bretton Z. H. Holt E. S. Alam R. Morton M. P. (2018). A novel method for chronic social defeat stress in female mice. *Neuropsychopharmacology* 43 1276–1283. 10.1038/npp.2017.259 29090682 PMC5916350

[B43] Hatzigiakoumis D. S. Martinotti G. Di Giannantonio M. Janiri L. (2011). Anhedonia and substance dependence: clinical correlates and: treatment options. *Front. Psychiatry* 2:10. 10.3389/fpsyt.2011.00010 21556280 PMC3089992

[B44] Heshmati M. Russo S. J. (2015). Anhedonia and the brain reward circuitry in depression. *Curr. Behav. Neurosci. Reports* 2 146–153. 10.1007/s40473-015-0044-3 26525751 PMC4626008

[B45] Hollon N. G. Burgeno L. M. Phillips P. E. M. (2015). Stress effects on the neural substrates of motivated behavior. *Nat. Neurosci.* 18 1405–1412. 10.1038/nn.4114 26404715 PMC4721524

[B46] Iñiguez S. D. Flores-Ramirez F. J. Riggs L. M. Alipio J. B. Garcia-Carachure I. Hernandez M. A. (2018). Vicarious social defeat stress induces depression-related outcomes in female mice. *Biol. Psychiatry* 83 9–17. 10.1016/j.biopsych.2017.07.014 28888327 PMC5730407

[B47] Kobrin K. L. Moody O. Arena D. T. Moore C. F. Heinrichs S. C. Kaplan G. B. (2016). Acquisition of morphine conditioned place preference increases the dendritic complexity of nucleus accumbens core neurons. *Addict. Biol.* 21 1086–1096. 10.1111/adb.12273 26096355

[B48] Kokane S. S. Perrotti L. I. (2020). Sex differences and the role of estradiol in mesolimbic reward circuits and vulnerability to cocaine and opiate addiction. *Front. Behav. Neurosci.* 14:74. 10.3389/FNBEH.2020.00074/BIBTEX PMC7251038 32508605

[B49] Koob G. F. (2020). Neurobiology of opioid addiction: opponent process, Hyperkatifeia, and negative reinforcement. *Biol. Psychiatry* 87 44–53. 10.1016/J.BIOPSYCH.2019.05.023 31400808

[B50] Krishnan V. Han M.-H. H. Graham D. L. Berton O. Renthal W. Russo S. J. (2007). Molecular adaptations underlying susceptibility and resistance to social defeat in brain reward regions. *Cell* 131 391–404. 10.1016/j.cell.2007.09.018 17956738

[B51] Lee C. W. S. Ho I. K. (2013). Sex differences in opioid analgesia and addiction: interactions among opioid receptors and estrogen receptors. *Mol. Pain* 9:45. 10.1186/1744-8069-9-45 24010861 PMC3844594

[B52] Luo H. Wu P. F. Cao Y. Jin M. Shen T. T. Wang J. (2020). Angiotensin-converting enzyme inhibitor rapidly ameliorates depressive-type behaviors *via* bradykinin-dependent activation of mammalian target of Rapamycin complex 1. *Biol. Psychiatry* 88 415–425. 10.1016/J.BIOPSYCH.2020.02.005 32220499

[B53] Lynch W. J. Carroll M. E. (1999). Sex differences in the acquisition of intravenously self-administered cocaine and heroin in rats. *Psychopharmacology (Berl.)* 144 77–82. 10.1007/S002130050979 10379627

[B54] Matsubara T. Matsuo K. Nakashima M. Nakano M. Harada K. Watanuki T. (1999). Morphine alters the structure of neurons in the nucleus accumbens and neocortex of rats. *Synapse* 85 160–162. 10.1016/j.neuroimage.2013.04.098 10400894

[B55] McHugh R. K. DeVito E. E. Dodd D. Carroll K. M. Potter J. S. Greenfield S. F. (2013). Gender differences in a clinical trial for prescription opioid dependence. *J. Subst. Abuse Treat.* 45 38–43. 10.1016/J.JSAT.2012.12.007 23313145 PMC3626739

[B56] Meng Y. Zhang Y. Tregoubov V. Falls D. L. Jia Z. (2003). Regulation of spine morphology and synaptic function by LIMK and the actin cytoskeleton. *Rev. Neurosci.* 14, 233–240. 10.1515/REVNEURO.2003.14.3.233 14513866

[B57] Miczek K. A. Nikulina E. M. Shimamoto A. Covington H. E. (2011). Escalated or suppressed cocaine reward, tegmental BDNF, and accumbal dopamine caused by episodic versus continuous social stress in rats. *J. Neurosci.* 31 9848–9857. 10.1523/JNEUROSCI.0637-11.2011 21734276 PMC3144494

[B58] Monroe S. Radke A. (2021). Aversion-resistant fentanyl self-administration in mice. *Psychopharmacology* 238, 699–710. 10.1007/s00213-020-05722-6 33226446 PMC7914171

[B59] Morais-Silva G. Nam H. Campbell R. Basu M. Pagliusi M. Fox M. E. (2021). Activation of Npas1-neurons in the ventral Pallidum mediates stress susceptibility. *bioRxiv* [preprint]. 10.1101/2021.10.27.466188

[B60] Nakayama A. Y. Harms M. B. Luo L. (2000). Small GTPases Rac and Rho in the maintenance of dendritic spines and branches in hippocampal pyramidal neurons. *J. Neurosci.* 20 5329–5338. 10.1523/jneurosci.20-14-05329.2000 10884317 PMC6772334

[B61] Negishi M. Katoh H. (2005). Rho family GTPases and dendrite plasticity. *Neuroscientist* 11 187–191. 10.1177/1073858404268768 15911868

[B62] Neisewander J. L. Peartree N. A. Pentkowski N. S. (2012). Emotional valence and context of social influences on drug abuse-related behavior in animal models of social stress and prosocial interaction. *Psychopharmacology (Berl)* 224 33–56. 10.1007/s00213-012-2853-3 22955569 PMC4071609

[B63] Newey S. E. Velamoor V. Govek E. E. Van Aelst L. (2005). Rho GTPases, dendritic structure, and mental retardation. *J. Neurobiol.* 64 58–74. 10.1002/neu.20153 15884002

[B64] Newman E. L. Leonard M. Z. Arena D. T. de Almeida R. M. M. Miczek K. A. (2018). Social defeat stress and escalation of cocaine and alcohol consumption: focus on CRF. *Neurobiol. Stress* 9 151–165. 10.1016/j.ynstr.2018.09.007 30450381 PMC6236516

[B65] Pal A. Das S. (2013). Chronic morphine exposure and its abstinence alters dendritic spine morphology and upregulates Shank1. *Neurochem. Int.* 62 956–964. 10.1016/j.neuint.2013.03.011 23538264

[B66] Phillips A. McGovern D. Lee S. Ro K. Huynh D. Elvig S. (2020). Oral prescription opioid-seeking behavior in male and female mice. *Addict. Biol.* 25:e12828. 10.1111/adb.12828 31489746

[B67] Ramsey N. F. Van Ree J. M. (1993). Emotional but not physical stress enhances intravenous cocaine self-administration in drug-naive rats. *Brain Res.* 608 216–222. 10.1016/0006-8993(93)91461-Z 8495356

[B68] Rigoni D. Avalos M. P. Boezio M. J. Guzmán A. S. Calfa G. D. Perassi E. M. (2021). Stress-induced vulnerability to develop cocaine addiction depends on cofilin modulation. *Neurobiol. Stress* 15:100349. 10.1016/J.YNSTR.2021.100349 34169122 PMC8209265

[B69] Robinson T. E. Gorny G. Savage V. R. Kolb B. (2002). Widespread but regionally specific effects of experimenter- versus self-administered morphine on dendritic spines in the nucleus accumbens, hippocampus, and neocortex of adult rats. *Synapse* 46 271–279. 10.1002/syn.10146 12373743

[B70] Sapolsky R. M. (2015). Stress and the brain: individual variability and the inverted-U. *Nat. Neurosci.* 18 1344–1346. 10.1038/nn.4109 26404708

[B71] Serdarevic M. Striley C. W. Cottler L. B. (2017). Sex differences in prescription opioid use. *Curr. Opin. Psychiatry* 30 238–246. 10.1097/YCO.0000000000000337 28426545 PMC5675036

[B72] Shaham Y. Alvares K. Nespor S. M. Grunberg N. E. (1992). Effect of stress on oral morphine and fentanyl self-administration in rats. *Pharmacol. Biochem. Behav.* 41 615–619. 10.1016/0091-3057(92)90382-p 1584842

[B73] Sinha R. (2008). Chronic stress, drug use, and vulnerability to addiction. *Ann. N. Y. Acad. Sci.* 1141 105–130. 10.1196/annals.1441.030 18991954 PMC2732004

[B74] Skolnick P. (2021). Treatment of overdose in the synthetic opioid era. *Pharmacol. Ther.* 108019. 10.1016/J.PHARMTHERA.2021.108019 34637841

[B75] Spiga S. Puddu M. C. Pisano M. Diana M. (2005). Morphine withdrawal-induced morphological changes in the nucleus accumbens. *Eur. J. Neurosci.* 22 2332–2340. 10.1111/j.1460-9568.2005.04416.x 16262671

[B76] Takahashi A. Chung J. R. Zhang S. Zhang H. Grossman Y. Aleyasin H. (2017). Establishment of a repeated social defeat stress model in female mice. *Sci. Rep.* 7:12838. 10.1038/s41598-017-12811-8 28993631 PMC5634448

[B77] Tidey J. W. Miczek K. A. (1997). Acquisition of cocaine self-administration after social stress: role of accumbens dopamine. *Psychopharmacology (Berl.)* 130 203–212. 10.1007/s002130050230 9151353

[B78] Towers E. Tunstall B. McCracken M. Vendruscolo L. Koob G. (2019). Male and female mice develop escalation of heroin intake and dependence following extended access. *Neuropharmacology* 151, 189–194. 10.1016/j.neuropharm.2019.03.019 30880124 PMC9345532

[B79] Venniro M. Russell T. I. Zhang M. Shaham Y. (2019). Operant social reward decreases incubation of heroin craving in male and female rats. *Biol. Psychiatry* 86 848–856. 10.1016/j.biopsych.2019.05.018 31326085 PMC8383184

[B80] Venniro M. Zhang M. Caprioli D. Hoots J. K. Golden S. A. Heins C. (2018). Volitional social interaction prevents drug addiction in rat models. *Nat. Neurosci.* 21 1520–1529. 10.1038/S41593-018-0246-6 30323276 PMC7386559

[B81] Warren B. L. Sial O. K. Alcantara L. F. Greenwood M. A. Brewer J. S. Rozofsky J. P. (2014). Altered gene expression and spine density in nucleus accumbens of adolescent and adult male mice exposed to emotional and physical stress. *Dev. Neurosci.* 36 250–260. 10.1159/000362875 24943326 PMC4125435

[B82] Warren B. L. Vialou V. F. Iñiguez S. D. Alcantara L. F. Wright K. N. Feng J. (2013). Neurobiological sequelae of witnessing stressful events in adult mice. *Biol. Psychiatry* 73 7–14. 10.1016/j.biopsych.2012.06.006 22795644 PMC3498570

[B83] Wemm S. E. Sinha R. (2019). Drug-induced stress responses and addiction risk and relapse. *Neurobiol. Stress* 10:100148. 10.1016/j.ynstr.2019.100148 30937354 PMC6430516

[B84] Yap J. J. Miczek K. A. (2007). Social defeat stress, sensitization, and intravenous cocaine self-administration in mice. *Psychopharmacology (Berl.)* 192 261–273. 10.1007/s00213-007-0712-4 17297635

